# MRI-Fusion Targeted vs. Systematic Prostate Biopsy–How Does the Biopsy Technique Affect Gleason Grade Concordance and Upgrading After Radical Prostatectomy?

**DOI:** 10.3389/fsurg.2019.00055

**Published:** 2019-09-18

**Authors:** Jessica Rührup, Felix Preisser, Lena Theißen, Mike Wenzel, Frederik C. Roos, Andreas Becker, Luis A. Kluth, Boris Bodelle, Jens Köllermann, Felix K. H. Chun, Philipp Mandel

**Affiliations:** ^1^Department of Urology, University Clinic Frankfurt, Frankfurt, Germany; ^2^Department of Radiology, University Clinic Frankfurt, Frankfurt, Germany; ^3^Department of Pathology, University Clinic Frankfurt, Frankfurt, Germany

**Keywords:** prostate cancer, mpMRI, biopsy, radical prostatectomy, Gleason upgrading, concordance

## Abstract

**Introduction:** MRI-targeted biopsy (TB) increases overall prostate-cancer (PCa) detection-rates and decreases the risk of insignificant PCa detection. However, the impact of these findings on the definite pathology after radical prostatectomy (RP) is under debate.

**Materials and Methods:** Between 01/2014 and 12/2018, 366 patients undergoing prostate biopsy and RP were retrospectively analyzed. The correlation between biopsy Gleason-score (highest Gleason-score in a core) and the RP Gleason-score in patients undergoing systematic biopsy (SB-group) (*n* = 221) or TB+SB (TB-group, *n* = 145) was tested using the ISUP Gleason-group grading (GGG, scale 1–5). Sub analyses focused on biopsy GGG 1 and GGG ≥ 2.

**Results:** Proportions of biopsy GGG 1–5 in the SB-group and TB-group were 24.4, 37.6, 19, 10.9, 8.1% and 13.8, 43.4, 24.2, 13.8, 4.8%, respectively (*p* = 0.07). Biopsy and pathologic GGG were concordant in 108 of 221 (48.9%) in SB- and 74 of 145 (51.1%) in TB-group (*p* = 0.8). Gleason upgrading was recorded in 33.5 and 31.7% in SB- vs. TB-group (*p* = 0.8). Patients with biopsy GGG 1 undergoing RP showed an upgrading in 68.5%(37/54) in SB- and 75%(15/20) in TB-group (*p* = 0.8). In patients with biopsy GGG ≥ 2 concordance increased for both biopsy approaches (54.5 vs. 55.2% for SB- vs. TB-group, *p* = 0.9).

**Discussion:** Irrespective of differences in PCa detection-rates between TB- and SB-groups, no significant differences in GGG concordance and upgrading between patients of both groups undergoing biopsy, followed by RP, were recorded. Concordance rates increased in men with biopsy GGG ≥ 2. TB seems to detect more patients with PCa without a difference in concordance with final pathology.

## Introduction

Prostate cancer (PCa) represents a frequently diagnosed cancer in Western countries, with a prevalence of 50–65% in men aged >60 years (1). To further improve survival in patients with PCa, it is important to diagnose PCa in localized, potential curable stages (2). In current clinical practice, detection of PCa consists of a randomized, systematic biopsy (SB) of the prostate in patients with elevated PSA-levels or suspicion of PCa at digital rectal examination (DRE) (3–5). The use of multiparametric magnetic resonance imaging (mpMRI) and MRI-targeted biopsy (TB) to improve PCa detection is a frequently debated topic (6–9), especially since current multicentric randomized trials, like the “Prostate evaluation for clinically important disease: sampling using image guidance or not?”–trial (PRECISION) and the “Diagnostic accuracy of multi-parametric MRI and TRUS biopsy in prostate cancer”-trial (PROMIS), showed the superiority of TB—by increasing the overall PCa detection rate and reducing the detection of insignificant tumors (e.g., Gleason 3+3) (10, 11). These results were recently confirmed by the “Use of prostate systematic and targeted biopsy on the basis of multiparametric MRI in biopsy-naive patients” study (MRI-first), where the added value of TB was 7.6 vs. 5.2% in SB, with only 5.6% of all patients who underwent TB showing insignificant PCa (12).

Many debates on the benefit of TB often broach the issue how these findings affect the definite pathology in specimen after radical prostatectomy (RP) (13, 14). However, the effect on Gleason concordance and up-/downgrading rates between biopsy and specimen are currently a matter of debate. Moreover, a precise diagnosis is crucial for optimal treatment decision-making. A possible impact of performing TB compared to SB might be a higher concordance rate due to a more representative biopsy in TB, or a lower concordance rate because of an overestimation especially of the Gleason-4 percentage in TB. Vice versa, there might be an underestimation of significant tumors in SB and a higher amount of upgrading at final pathology in patients undergoing SB.

The aim of the present study was to correlate the biopsy ISUP Gleason-group grading (GGG) and the GGG of the RP specimen, stratified by the approach of biopsy (SB vs. TB and SB). Subgroups focused on patients with the detection of low-risk PCa.

## Materials and Methods

### Patients

Based on our prospective institutional review-board approved database, 366 consecutive patients who underwent prostate biopsy and RP during the period between January 2014 and December 2018 were identified and included in our retrospective analysis.

All patients gave informed consent. Ethical approval was obtained from the local ethics committee at the University hospital of Frankfurt.

### Biopsy Criteria and Histology

All biopsies were taken with a transrectal approach under local anesthesia and antibiotic prophylaxis. Indication for SB was a suspicious PSA-level/rectal examination of the prostate, according to current guidelines (4, 15). SB was performed using a 12-core approach (SB-group). In patients with a PI-RADS-lesion ≥3 (according to the Prostate Imaging–Reporting and Data System “PI-RADS”-v2 classification) (16) in the mpMRI of the prostate, additional to the SB a TB was performed (TB-group). All mpMRI for targeted biopsies were reviewed by an internal specified radiologist.

All TBs were conducted as MRI-targeted ultrasound fusion biopsy using the “Ascendus Hi-Vision” system by Hitachi. There are different methods for performing TB. In our department we performed image-fusion TB; cognitive (mental) fusion TB and in-bore are therefore not subject of the present manuscript.”

The biopsy cores and specimen after RP were evaluated by dedicated uro-pathologists. The biopsy Gleason was defined as highest Gleason-score in at least one core and was reported using the ISUP Consensus Conference 2014 grading system (17).

### Statistical Analysis

Descriptive statistics included medians for continuous variables and frequencies and proportions for categorical variables. Differences were analyzed with the use of two-group mean-comparison *t*-test, the Kruskall-Wallis test and the Pearson's chi-squared test. Univariable analysis were performed to compare concordance, upgrading and downgrading rates of the ISUP Gleason-group grading (GGG, scale 1–5) of biopsy and specimen using SPSS software (PASW Statistics 18, Software by IBM, Ehningen, Germany). A *p*-value of 0.05 or lower was considered statistically significant.

## Results

**Figure 1:**
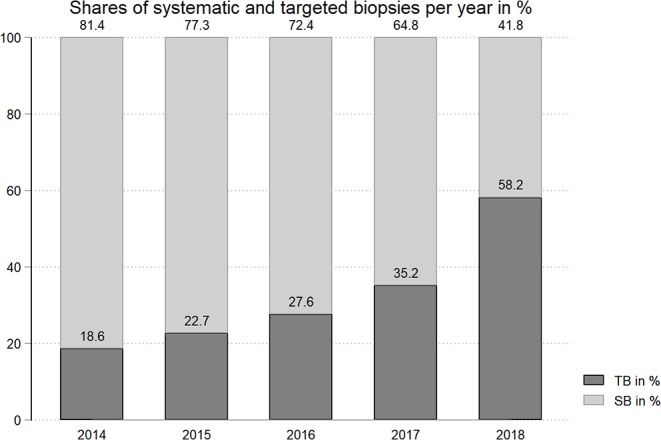
Distribution of SB vs. TB at our institution over the years (2014–2018) in percent.

Descriptive characteristics of the entire patient cohort and stratified by biopsy approach are depicted in Table 1. Overall, 221 patients underwent SB (60.4%) vs. 145 patients (39.6%) who constituted the TB-group. Proportion of patients undergoing TB increased over the years (Figure 1). There were no statistically significant differences regarding age, prostate volume, and median PSA-levels between the two groups (Table 1). The distribution of PI-RADS 3–5 in patients of the TB-group was 13.1, 45.5, and 41.4%, respectively. The total proportions of biopsy GGG 1–5 in patients with SB were 24.4, 37.6, 19.0, 10.9, and 8.1% and 13.8, 43.4, 24.2, 13.8, and 4.8% in patients of the TB-group, respectively (*p* = 0.07). At final pathology GGG 1–5 in men of the SB-group were 10.0, 54.7, 12.7, 9.5, and 13.1%, and 5.5, 49.7, 23.4, 7.6, and 13.8% of the TB-group (*p* = 0.06).

**Table 1:** Descriptive characteristics of patients that underwent prostate biopsy between 2014 and 2018, stratified according to systematic biopsy (SB) vs. targeted biopsy (TB).

	**All patients** ***n* = 366**	**SB group** ***n* = 221**	**TB group** ***n* = 145**	***P*-value**
Age, years, median (interquartile range)	67.2 (62.1–71.5)	67.0 (62.1–70.7)	67.5 (62.1–72.6)	0.2
Prostate volume, ml, median (interquartile range)	37 (28–50)	35 (28–50)	40 (30–50)	0.3
PSA-value prior to biopsy, ng/ml, median (interquartile range)	8.4 (5.8–13.1)	8.7 (5.9–14.0)	8.2 (5.6–12.7)	0.3
**pT stage**, ***n*** **(%), specimen**				
pT2	*n* = 220 (60.1)	*n* = 132 (59.7)	*n* = 88 (60.7)	0.02
pT3a	*n* = 87 (23.8)	*n* = 45 (20.4)	*n* = 42 (29.0)	
≥pT3b	*n* = 59 (16.1)	*n* = 44 (19.9)	*n* = 15 (10.3)	
**R stage**, ***n*** **(%)**				
R0	*n* = 281 (76.8)	*n* = 165 (74.7)	*n* = 116 (80)	0.3
R1	*n* = 85 (23.2)	*n* = 56 (25.3)	*n* = 29 (20)	
**pN stage**, ***n*** **(%)**				
pN0/pNx	*n* = 323 (88.3)	*n* = 190 (86)	*n* = 133 (91.7)	0.1
pN1	*n* = 42 (11.7)	*n* = 31 (14)	*n* = 12 (8.3)	
**PI-RADS**, ***n*** **(%)**				
PI-RADS 3			*n* = 19 (13.1)	
PI-RADS 4			*n* = 66 (45.5)	
PI-RADS 5			*n* = 60 (41.4)	
**GGG biopsy**, ***n*** **(%)**				
1 (3+3)	*n* = 74 (20.2)	*n* = 54 (24.4)	*n* = 20 (13.8)	0.07
2 (3+4)	*n* = 146 (39.9)	*n* = 83 (37.6)	*n* = 63 (43.4)	
3 (4+3)	*n* = 77 (21)	*n* = 42 (19)	*n* = 35 (24.2)	
4 (4+4, 3+5, 5+3)	*n* = 44 (12)	*n* = 24 (10.9)	*n* = 20 (13.8)	
5 (5+4, 4+5, 5+5)	*n* = 25 (6.8)	*n* = 18 (8.1)	*n* = 7 (4.8)	
**GGG specimen**, ***n*** **(%)**				
1 (3+3)	*n* = 30 (8.2)	*n* = 22 (10)	*n* = 8 (5.5)	0.6
2 (3+4)	*n* = 193 (52.7)	*n* = 121 (54.7)	*n* = 72 (49.7)	
3 (4+3)	*n* = 62 (16.9)	*n* = 28 (12.7)	*n* = 34 (23.4)	
4 (4+4, 3+5, 5+3)	*n* = 32 (8.7)	*n* = 21 (9.5)	*n* = 11 (7.6)	
5 (5+4, 4+5, 5+5)	*n* = 49 (13.4)	*n* = 29 (13.1)	*n* = 20 (13.8)	

Biopsy and pathologic GGG were concordant in 108 out of 221 (48.9%) men of the SB- and in 74 out of 145 (51.1%) of the TB-group (*p* = 0.8) (Tables 2A,B, 3A). Gleason upgrading/downgrading of at least one GGG was recorded in 33.5/17.6% patients of the SB-, and in 31.7/17.2% of the TB-group, respectively (all *p* > 0.05).

**TABLE 2A:** Biopsy vs. specimen Gleason Grade Groups (GGG) in the subgroup of patients with systematic biopsy (SB).

**GGG Biopsy, *n***	**GGG specimen**					
	**GGG 1**	**GGG 2**	**GGG 3**	**GGG 4**	**GGG 5**	**Total**
GGG 1	17	33	0	4	0	54
GGG 2	4	58	13	4	4	83
GGG 3	0	20	12	4	6	42
GGG 4	1	8	1	8	6	24
GGG 5	0	2	2	1	13	18
Total	22	121	28	21	29	221

**TABLE 2B:** Biopsy vs. specimen Gleason Grade Groups (GGG) in the subgroup of patients with targeted biopsy (TB).

**GGG Biopsy, *n***	**GGG specimen**					
	**GGG 1**	**GGG 2**	**GGG 3**	**GGG 4**	**GGG 5**	**Total**
GGG 1	5	12	2	0	1	20
GGG 2	3	42	11	3	4	63
GGG 3	0	10	18	3	4	35
GGG 4	0	7	3	4	6	20
GGG 5	0	1	0	1	5	7
Total	8	72	34	11	20	145

**TABLE 3A:** Concordance, upgrading and downgrading of biopsy compared to specimen Gleason Grade Groups (GGG) stratified by biopsy approach.

	**Entire cohort**	**SB group**	**TB group**	***P*-value**
Concordance	*n* = 182 (49.7%)	*n* = 108 (48.9%)	*n* = 74 (51.1%)	0.77
Upgrading	*n* = 120 (32.8%)	*n* = 74 (33.5%)	*n* = 46 (31.7%)	0.81
Downgrading	*n* = 64 (17.5%)	*n* = 39 (17.6%)	*n* = 25 (17.2%)	0.99
Total	*n* = 366 (100%)	*n* = 221 (100%)	*n* = 145 (100%)	

*SB group, systematic biopsy; TB group, targeted biopsy and systematic biopsy*.

In patients with biopsy GGG 1 exclusively, 70.3% of all patients showed an upgrading in final pathology. Upgrading rates for patients of the SB- vs. TB-group showed an insignificant trend for a higher risk of upgrading in the TB-group (68.5 vs. 75%, *p* = 0.8, Table 3B). Downgrading to GGG 1 in final pathology was very rare and occurred only in eight patients (five patients of SB-group vs. three patients of TB-group).

**TABLE 3B:** Concordance and upgrading of patients with biopsy Gleason Grade Group (GGG) 1 compared to specimen GGG stratified by biopsy approach.

	**Entire cohort**	**SB group**	**TB group**	***P*-value**
Concordance	*n* = 22 (29.7%)	*n* = 17 (31.5%)	*n* = 5 (25%)	0.8
Upgrading	*n* = 52 (70.3%)	*n* = 37 (68.5%)	*n* = 15 (75%)	0.8
Total	*n* = 74 (100%)	*n* = 54 (100%)	*n* = 20 (100%)	

*SB group, systematic biopsy; TB group, targeted biopsy and systematic biopsy*.

In patients with biopsy GGG ≥ 2 concordance rates in both groups increased to 54.5 vs. 55.2% (for SB- vs. TB-group, *p* = 0.9, Table 3C).

**TABLE 3C:** Concordance, upgrading, and downgrading of patients with biopsy GGG ≥ 2 compared to specimen GGG stratified by biopsy approach.

	**Entire cohort**	**SB group**	**TB group**	***P*-value**
Concordance	*n* = 160 (54.8%)	*n* = 91 (54.5%)	*n* = 69 (55.2%)	0.9
Upgrading	*n* = 68 (23.3%)	*n* = 37 (22.2%)	*n* = 31 (24.8%)	0.7
Downgrading	*n* = 64 (21.9%)	*n* = 39 (23.3%)	*n* = 25 (20.0%)	0.6
Total	*n* = 292 (100%)	*n* = 167 (100%)	*n* = 125 (100%)	

## Discussion

In contemporary years, mpMRI of the prostate is becoming an integrative part in the diagnostic workup of PCa (10, 18, 19). Several prospective trials demonstrated that TB can increase PCa detection rates, especially the detection rate of clinically significant PCa (GGG ≥ 2), while lowering the detection rate of low-risk-PCa (9, 11, 20). Despite that, little is known about the concordance of biopsy GGG and GGG at RP specimen. Therefore, we investigated concordance and upgrading rates of GGG in patients who underwent SB (SB-group) vs. SB+TB (TB-group) and the specimen GGG after RP. Our study demonstrated several noteworthy findings which are discussed in the following.

Within the current analysis, based on 366 patients, no significant differences in concordance, upgrading, and downgrading rates in patients of the SB- compared to the TB-group were identified. Concordance increased for both biopsy approaches in the subgroup of patients with biopsy GGG ≥ 2. Thereby, both biopsy approaches represent the “true” GGG of the RP specimen in the same way. Patients of the TB-group in general did not show a more representative biopsy (or a high grade of downgrading as reported by some authors) (20) and SB did not underestimate significant tumors in a higher number of cases. However, besides the above mentioned higher detection rate, the availability of an mpMRI of the prostate has several other advances for patients undergoing RP, which were not tested in the present study (e.g., improvement of local T-stage evaluation, improvement of local therapeutic decision regarding nerve sparing) (21, 22). Despite the known advantages of TB, one has to keep in mind that urologists who perform TB have to undergo a certain learning curve (23).

In contrast to our findings, some authors found the use of TB to be associated with lower rates of Gleason upgrading and higher concordance (13, 14, 24, 25). The largest available study is based on a multicentric cohort published in 2019 by Diamand et al. comparing the concordance rates of TB, SB, and a combination of TB and SB in 443 men undergoing RP (24). The authors described a concordance rate of patients undergoing SB alone of 49.4, 43.1 % of patients had an upgrading at final pathology and in 7.4% a GGG downgrading was recorded. Patients who underwent SB and TB had a concordance rate of 63.2% and upgrading and downgrading rates were 23.9 and 12.9%, respectively. Whereas, concordance in the SB-group was comparable to our data (49.4 vs. 48.9%), concordance in patients undergoing SB and TB was higher compared to the present study (63.2 vs. 51.1%). While patient characteristics (such as age, prostate volume, PSA) were similar, patients analyzed by Diamand et al. had significantly lower biopsy GGG, which might have affected their results. In our series only 8.2% of the patients underwent RP with a GGG 1, but 26.6% of the men analyzed by Diamand et al.–therefore our patient cohort rather represents a contemporary “real-life” cohort, as a stage migration toward more aggressive PCa in patients undergoing RP is described in literature (26). When concentrating only on the results of patients with a GGG ≥ 2, as these patients also likely benefit from a RP, concordance in the TB-group increased in our cohort to 55.2%, which is almost identical to the rate published by Diamand et al. for this subgroup (56.7%). Moreover, patients in our cohort had more advanced disease (e.g., 8.5% in Diamand's cohort vs. 16.1% of our patients showing a pathologic stage ≥pT3b, *p* = 0.02), which might have especially positively affected the results in the SB-group (19.9% ≥pT3b) in terms of an increase in concordance, as it might be more likely to obtain a randomized biopsy with the “true” GGG in patients with more advanced tumor.

Interestingly, upgrading rates in patients with biopsy GGG 1 had a trend to be higher for patients of the TB- compared to the SB-group. These results corroborate a study by Kayano et al. (14). At final pathology, patients with a GGG 1 in the TB-group were upgraded in 75% compared to 65.4% of the SB-group, which is almost identical with the upgrading rates from our series (75 vs. 68.5%) in this subgroup. However, in both studies, this subgroup consisted only of a small number of patients which might lead to a selection bias.

To further improve the concordance in patients undergoing TB, one should always simultaneously perform a SB (27, 28). Arsov et al. showed a reduced risk of Gleason upgrading when performing a combined SB and TB compared to SB or TB alone (27). Moreover, upgrading rates in patients undergoing TB also seem to be dependent of the extent of TB. Calio et al. prospectively analyzed the data of 208 patients who were divided into two groups, both undergoing TB and SB. One group received a saturation biopsy of the index lesion in mpMRI, and the other group a non-saturated biopsy. The results showed significantly fewer upgrading rates in the saturated lesion group as well as higher concordance rates (13).

The present study has several limitations. First and foremost, our manuscript is based on a retrospective analysis of only one tertiary center. Even though comparable studies do not rely on larger sample sizes, our study is limited to the rather small cohort of 366 patients. Moreover, all our patients in the TB-group underwent simultaneous TB and SB and therefore we cannot measure a potential benefit of SB during TB. The decision to perform SB or a combined SB and TB was made according to current guidelines and to the discretion of the treating urologist and the patient. This might lead to a potential inclusion bias.

Finally, not all patients with histologic confirmed PCa at biopsy underwent RP, which might have led to a potential selecting bias, especially in the subgroup of patients with lower GGG.

In conclusion, irrespective of differences in PCa detection rates between SB and TB, no significant differences in GGG concordance and upgrading rates between patients of the SB- vs. TB-group, followed by RP, were detected. These results were similar in the entire cohort and in patients within the analyzed subgroups (GGG 1 and GGG ≥ 2), whereas concordance rates of the TB-group increased with higher GGG. Therefore, in general TB detects more patients with PCa without a difference in concordance rate at final pathology.

## Data Availability

The datasets generated for this study are available on request to the corresponding author.

## Ethics Statement

This studies involving human participants were reviewed and approved by Ethik-Kommission Fachbereich Medizin, Goethe-Universität Frankfurt. The patients/participants provided their written informed consent to participate in this study.

## Author Contributions

LT and MW: acquisition of the analyzed data. FP: acquisition and statistical analysis of data. FR, AB, and LK: final approval of the version to be published and advises during daily work-routine. BB and JK: acquisition of data and final approval of the version to be published. FC: substantial contributions to the conception of the work and final approval of the version to be published. PM: substantial contributions to the conception of the work, revising the manuscript critically for the intellectual content, interpretation of data, and final approval of the version to be published. JR: acquisition, analysis and interpretation of data, substantial contributions to the conception of the work, drafting the work, and revising the work for the intellectual content. All authors complied with all aspects of the work. They ensure that questions related to the accuracy of the work are adequately discussed and solved.

### Conflict of Interest Statement

The authors declare that the research was conducted in the absence of any commercial or financial relationships that could be construed as a potential conflict of interest.
